# Editorial Comment: Environmental Impact of Prostate Magnetic Resonance Imaging and Transrectal Ultrasound Guided Prostate Biopsy

**DOI:** 10.1590/S1677-5538.IBJU.2023.03.02

**Published:** 2023-04-15

**Authors:** Lorenzo Storino Ramacciotti, Masatomo Kaneko, Michael Eppler, Giovanni E. Cacciamani, Andre Luis Abreu

**Affiliations:** 1 University of Southern California Institute of Urology USA University of Southern California - USC, Institute of Urology, Center for Image-Guided Surgery, Focal Therapy and Artificial Intelligence for Prostate Cancer, Keck School of Medicine, University of Southern California, Los Angeles, CA, USA;; Center for Image-Guided Surgery, Focal Therapy and Artificial Intelligence for Prostate Cancer Keck School of Medicine University of Southern California Los Angeles CA USA; 2 Department of Urology Graduate School of Medical Science Kyoto Prefectural University of Medicine Kyoto Kyoto Japan Department of Urology, Graduate School of Medical Science, Kyoto Prefectural University of Medicine, Kyoto, Kyoto, Japan;; 3 Department of Radiology Keck School of Medicine University of Southern California Los Angeles CA USA Department of Radiology, Keck School of Medicine, University of Southern California, Los Angeles, CA, USA

Michael S Leapman ^1^, Cassandra L Thiel ^2^, Ilyssa O Gordon ^3^, Adam C Nolte ^4^, Aaron Perecman ^5^, Stacy Loeb ^6^, Michael Overcash ^7^, Jodi D Sherman ^8^ *Eur Urol. 2023 Jan 10;S0302-2838(22)02857-3*

## COMMENT

The concept of sustainability in medical practice involves minimizing the negative impact of healthcare activities on the environment without compromising patient care (1). This includes reducing waste, energy consumption, and greenhouse gas (GHG) emissions. Leapman et al. should be congratulated for their study, as it contributes to understanding the importance of the carbon footprint of prostate magnetic resonance imaging (MRI) and biopsy - both critical components of prostate cancer diagnosis and treatment (2).

The study involved academic medical centers in the USA, outpatient urology clinics, and health care facilities. It estimated the GHG emissions (CO2 equivalents) and equivalents of coal and gasoline burned in five clinical scenarios: I) multiparametric MRI (mpMRI) of the prostate with targeted and systematic biopsies (baseline); II) mpMRI with targeted biopsy cores only; III) systematic biopsy without MRI; IV) mpMRI with systematic biopsy only; V) biparametric MRI (bpMRI) with targeted and systematic biopsies. The data on materials and energy consumption, patient and staff travel were analyzed for each component (Steps) of the procedure, as follows: 1) pre-biopsy mpMRI; 2) Transrectal ultrasound (TRUS) and prostate biopsy in the outpatient clinic; 3) Pathology laboratory.

The results showed that the carbon footprint for a single patient undergoing mpMRI, TRUS with targeted and systematic prostate biopsy was 80.7 kg CO2, equivalent to burning 34.4 liters of gasoline or 40.5 kg of coal. Conversely, a systematic 12-core biopsy without mpMRI generated 36.2 kg CO2 equivalent and was the less ominous scenario for the environment. Using bpMRI instead of mpMRI with targeted and systematic biopsies resulted in a 10.7% reduction in GHG emissions. Energy consumption, which includes power and electricity usage, was identified as the leading contributor to GHG emissions, with staff travel being the second most significant contributor. Among the procedure Steps, the mpMRI had the greatest impact on the carbon footprint, and the mpMRI alone contributed 42.7 kg CO2e (54.3% of the baseline scenario). If MRI is performed as a triage strategy to select candidates for biopsy (avoid unnecessary biopsies) and limit sampling to MRI-targeted suspicious areas, the carbon emissions would be reduced by 1.4 million kg CO2e per 100,000 patients, equivalent to consuming 700,000 liters of gasoline. This would have a considerable environmental impact since it is estimated that the USA and Europe combined perform over 2 million prostate biopsies annually (3).

Although the study provides valuable insights into the carbon footprint of transrectal prostate biopsy, it has limitations. Indeed, it does not explore the potential differences in GHG emissions between transrectal and transperineal biopsy procedures and does not account for downstream infectious complications, hospitalizations, etc. (4). Additionally, it would be valuable to evaluate the potential advantages of performing a “One-Stop” and “RAPID” procedure that combines MRI and prostate biopsy on the same day (5, 6). This approach could significantly reduce patient and staff travel, resulting in substantial environmental benefits. Further improvements in MRI protocols, such as fast and bpMRI, and the integration of artificial intelligence (AI) algorithms could enhance MRI performance, address its limitations, and substantially decrease unnecessary prostate biopsies (7).

Overall, this study highlights the importance of sustainable solutions to reduce the carbon footprint in healthcare. An optimal pathway for sustainability associated with patient care would include: I) One-Stop bpMRI with fast protocols aided by AI; II) targeted biopsy exclusively; III) a transperineal approach performed under local anesthesia in an office-based setting. Further research is needed to establish sustainable solutions for reducing greenhouse gas emissions in prostate cancer management that do not compromise the individual yet minimize environmental impact while benefiting humankind.
